# Tri-methylation of ATF7IP by G9a/GLP recruits the chromodomain protein MPP8

**DOI:** 10.1186/s13072-018-0231-z

**Published:** 2018-10-04

**Authors:** Takeshi Tsusaka, Masaki Kikuchi, Tadahiro Shimazu, Takehiro Suzuki, Yoshihiro Sohtome, Mai Akakabe, Mikiko Sodeoka, Naoshi Dohmae, Takashi Umehara, Yoichi Shinkai

**Affiliations:** 1 Cellular Memory Laboratory, RIKEN Cluster for Pioneering Research, Wako, 351-0198 Japan; 2 Department of Diabetes, Endocrinology and Nutrition, Graduate School of Medicine, Kyoto University, Kyoto, 606-8507 Japan; 3 Laboratory for Epigenetics Drug Discovery, RIKEN Center for Biosystems Dynamics Research, Yokohama, 230-0045 Japan; 4 Biomolecular Characterization Unit, Technology Platform Division, RIKEN Center for Sustainable Resource Science, Wako, 351-0198 Japan; 5 Synthetic Organic Chemistry Laboratory, RIKEN Cluster for Pioneering Research, Wako, 351-0198 Japan; 6 RIKEN Center for Sustainable Resource Science, Wako, 351-0198 Japan

**Keywords:** G9a/Ehmt2, GLP/Ehmt1, ATF7IP, MPP8, Non-histone methylation, Transgene silencing, Lysine methylation

## Abstract

**Background:**

G9a and the related enzyme GLP were originally identified as histone lysine methyltransferases and then shown to also methylate several other non-histone proteins.

**Results:**

Here, we performed a comprehensive screen to identify their substrates in mouse embryonic stem cells (mESCs). We identified 59 proteins, including histones and other known substrates. One of the identified substrates, activating transcriptional factor 7-interacting protein 1 (ATF7IP), is tri-methylated at a histone H3 lysine 9 (H3K9)-like mimic by the G9a/GLP complex, although this complex mainly introduces di-methylation on H3K9 and DNA ligase 1 (LIG1) K126 in cells. The catalytic domain of G9a showed a higher affinity for di-methylated lysine on ATF7IP than LIG1, which may create different methylation levels of different substrates in cells. Furthermore, we found that M-phase phosphoprotein 8 (MPP8), known as a H3K9me3-binding protein, recognizes methylated ATF7IP via its chromodomain. MPP8 is also a known component of the human silencing hub complex that mediates silencing of transgenes via SETDB1 recruitment, which is a binding partner of ATF7IP. Although the interaction between ATF7IP and SETDB1 does not depend on ATF7IP methylation, we found that induction of SETDB1/MPP8-mediated reporter-provirus silencing is delayed in mESCs expressing only an un-methylatable mutant of ATF7IP.

**Conclusions:**

Our findings provide new insights into the roles of lysine methylation in non-histone substrates which are targeted by the G9a/GLP complex and suggest a potential function of ATF7IP methylation in SETDB1/MPP8-mediated transgene silencing.

**Electronic supplementary material:**

The online version of this article (10.1186/s13072-018-0231-z) contains supplementary material, which is available to authorized users.

## Background

Post-translational modifications (PTMs) of histones contribute to the change and maintenance of cellular functions through control of gene expression [1–3]. Lysine methylation is one of the most studied histone PTMs and occurs at multiple histone protein residues. Methylation of different lysine residues is catalyzed by different protein lysine methyltransferases (PKMTs) and has different roles in chromatin-coupled functions such as transcription. For example, H3K9 methylation is a well-conserved silencing modification induced by multiple enzymes such as SUV39H1 and SUV39H2 [4, 5], SETDB1 (also known as ESET) [6, 7], G9a (also known as EHMT2) [8, 9], GLP (also known as EHMT1) [10, 11], PRDM2 [12], and PRDM3 and PRDM16 [13] in mammals. Furthermore, these methyltransferases target distinct regions in the genome and establish a different methylation status in cellular H3K9. SUV39H1/2 and SETDB1 are responsible for tri-methylation at constitutive heterochromatic regions and euchromatic regions, respectively. In contrast, G9a and GLP mainly catalyze H3K9 di-methylation at a vast number of euchromatic regions. These H3K9 methylations are thought to repress transcription, but the detailed molecular mechanisms remain unclear [14].

G9a and GLP are SET-domain-type lysine methyltransferases that primarily exist as a heteromeric complex in vivo [11]. They play crucial roles in various biological processes such as mouse embryonic and germ cell development, immune functions, and behaviour [15]. However, the precise mechanisms of G9a/GLP-mediated regulation and the amount of H3K9 methyltransferase activity needed for full function are still unclear. Furthermore, G9a and GLP have been shown to methylate multiple proteins other than histones [16]. The biological significance of most of the non-histone protein methylations is still unknown. However, recent work has established that the G9a/GLP-mediated non-histone methylations are involved in gene expression and the maintenance of DNA methylation during DNA replication, independently from H3K9 methylation [17–19]. These findings let us reconsider the G9a/GLP roles yet to be distinguished as either H3K9 methylation-dependent or methylation-independent. To this end, a more comprehensive screen of non-histone G9a/GLP substrates would be worthful.

Several attempts have been made to identify methylated substrates of PKMTs by taking advantage of antibodies specific to methylated proteins, methyl-binding domains, or chemical probes [20]. Among them, the use of a *S*-adenosyl-l-methionine (SAM) surrogate, which contains an alkyne or azide moiety, has been established as a powerful approach that can be coupled with an affinity tagging via copper-catalyzed azide-alkyne cycloaddition (CuAAC) reaction (known as click chemistry) [21–24]. Propargylic *Se*-adenosyl-l-selenomethionine (ProSeAM, also called SeAdoYn) is a SAM analogue with an alkyne moiety and has been shown to be active for a variety of methyltransferases including G9a and GLP [22, 23]. We previously used ProSeAM to probe for protein methylations in cell lysates [25]. This allowed us to identify methylated substrates of a specific methyltransferase at the proteomic level. However, the previous protocol had some drawbacks, especially a potential diminishment or impairment of the detection of some substrates by pre-existing full or high methylation on the substrates of cell lysates.

In this study, we performed an improved comprehensive substrate screen for G9a/GLP using mouse embryonic stem cells (ESCs) deficient for *G9a* and *Glp* [26]. We found that several high-ranked candidate proteins have amino-acid sequences locally similar to the histone H3 N-terminus surrounding K9, which we refer to as H3K9-like sequences. We focused our attention on one of the identified G9a/GLP non-histone target candidates containing a H3K9-like sequence. The role of ATF7IP (also known as MCAF1 or mAM), a known SETDB1 binding partner involved in transcriptional regulation [27–31], methylation by G9a/GLP was examined.

## Results

### A methyl-donor analogue-based screen identified ATF7IP as a G9a/GLP substrate

**Fig. 1:**
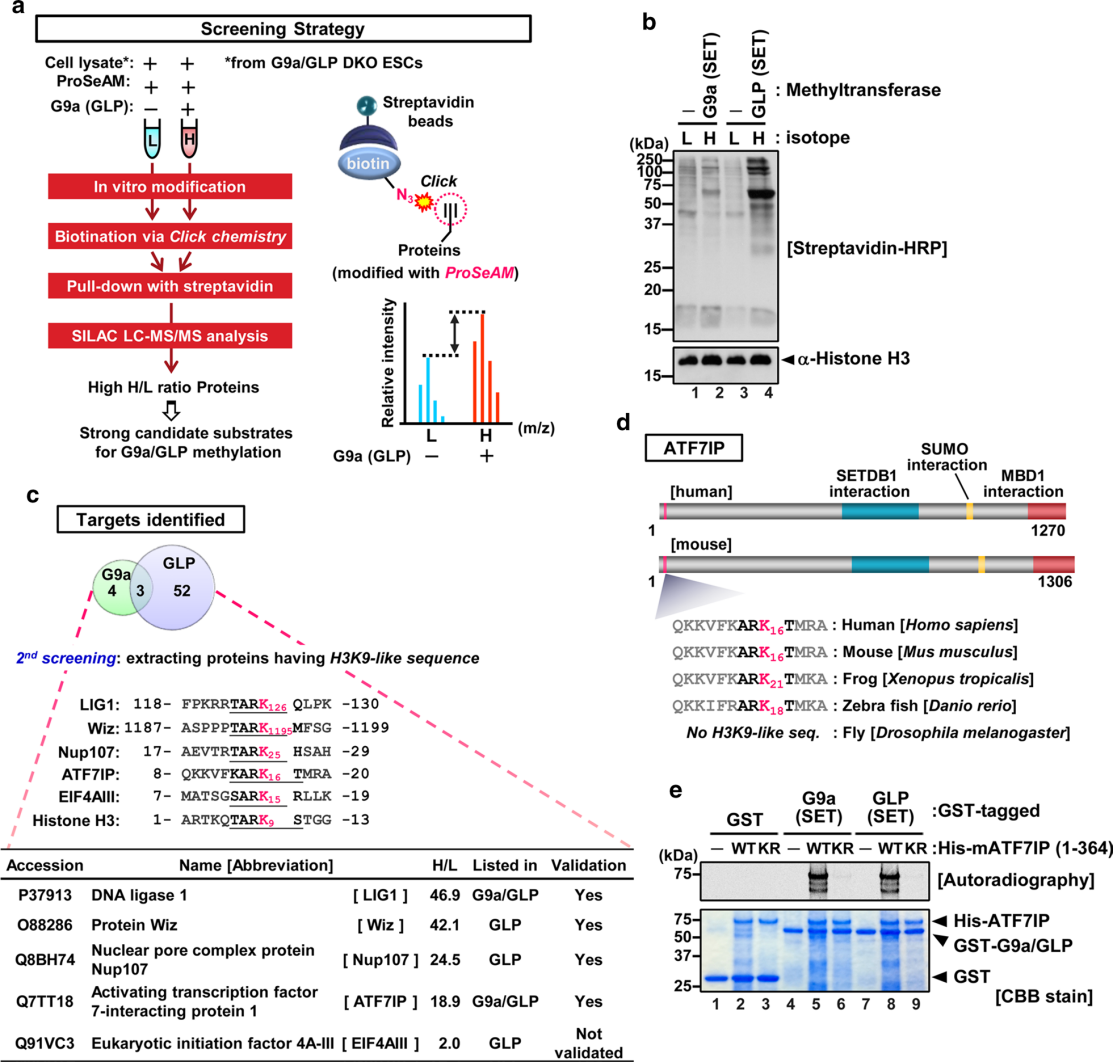
A methyl-donor analogue-based screen identified ATF7IP as a G9a/GLP substrate. **a** A scheme of ProSeAM-based screen of substrates for G9a or GLP methylation. L and H indicate light-(^12^C6) and heavy-(^13^C6) lysine labelling, respectively. **b** Western blot analysis with streptavidin-HRP shows a labelling of biotin tag to endogenous proteins in the cell lysates. Blot with anti-H3 antibody was used as a loading control. **c** A Venn diagram of identified proteins in the screen. Shared candidates were ATF7IP, LIG1, and ARFG2. Identified histone H3K9-like sequences within substrates were shown. Validation experiments were done in this study (ATF7IP and Nup107) or previous studies (Wiz and LIG1; [16, 19]). **d** A schematic of human and mouse ATF7IP protein. Known regions that interact with other proteins were indicated in colour. A histone H3K9-like sequence locates in the N-terminus of ATF7IP. The histone H3K9-like motif is conserved among vertebrates. **e** An in vitro methylation assay with recombinant ATF7IP protein (residues 1–364), GST-tagged SET-domain of G9a or GLP, and ^14^C-labelled SAM. An incorporation of the methyl-moiety was detected as an autoradiographic signal. WT and KR indicate wild-type and K16R mutant of ATF7IP, respectively

We have previously developed a screen for methylated substrates of a methyltransferase of interest [25]. This method takes advantage of a SAM analogue, ProSeAM. A wide spectrum of methyltransferases, including G9a, can use ProSeAM as a cofactor, resulting in propargylated substrates. The propargylated proteins are further conjugated with a biotin tag via a CuAAC reaction. To identify novel G9a and GLP targets in mESCs, we used cell lysates from *G9a* and *Glp* double knock-out (*G9a/GLP* DKO) mESCs, expecting an abrogation of pre-existing methylation by endogenous G9a and GLP in the substrate lysates (see scheme in Fig. 1a). The cell lysate of *G9a*/*Glp* DKO mESCs was incubated with GST-fused to either a G9a or GLP catalytic SET-domain in the presence of ProSeAM. After in vitro modification, reactants were subjected to the click reaction. The resulting propargylated molecules were conjugated with a biotin tag. Figure 1b shows the biotin labelling of various endogenous proteins by an addition of a G9a or GLP SET-domain (lanes 2 and 4), revealed by western blot analysis with streptavidin-HRP. We purified the biotinylated proteins using an avidin–biotin interaction and analyzed the purified proteins by liquid chromatography–tandem mass spectrometry (LC–MS/MS). The stable isotope labelling using amino acids with the cell culture (SILAC) method was combined with this proteomic analysis to make it more quantitative.

After employing an initial arbitrary cutoff of a H/L peak area’s ratio > 2 (H and L indicate samples with and without a methyltransferase, respectively), we identified 7 and 55 proteins as G9a and GLP target candidates, respectively (three of them overlapped and the full list is in Additional file 1: Table S1). They include histones H1 and H3 and known G9a or GLP non-histone substrates such as LIG1 and Wiz [16, 19]. To further narrow the potential candidates, we searched for the histone H3-like sequence (ARK) that seems to be the G9a/GLP target consensus sequence [16] in the identified candidate protein amino-acid sequences and found that 5 proteins possess such consensus sequence (Fig. 1c). These include LIG1, Wiz, nuclear pore complex protein Nup107 (Nup107), ATF7IP, and eukaryotic initiation factor 4A-III (eIF4AIII). Nup107 and eIF4AIII have not, so far, been reported as G9a/GLP targets. We prepared 3xFLAG-tagged Nup107 from transfected HEK293T cells and performed in vitro methylation of purified Nup107 with recombinant G9a or a GLP SET-domain in the presence of radioactive SAM. This result showed that Nup107 was methylated at its H3K9-like lysine (K25 for human) by G9a or GLP, at least in vitro (Additional file 2: Fig. S1).

Although it has been reported that ATF7IP contains the H3K9-like sequence at its N-terminus (Fig. 1d), and that the corresponding peptide is methylated by G9a [32, 33], the details of ATF7IP methylation and its biological function have not yet been investigated. The H3K9-like motif within ATF7IP is highly conserved among species including mammals, frogs, and zebra fish, but excluding flies, and lysine 16 (K16) of human/mouse ATF7IP corresponds to lysine 9 of histone H3. We questioned whether the ATF7IP protein could be methylated by G9a and/or GLP and, if so, whether the methylation occurs at the H3K9-like lysine K16. For this, the mouse ATF7IP N-terminus portion (residues 1–364) was produced in bacteria and incubated with a catalytic SET-domain of G9a or GLP in the presence of radioactive ^14^C-SAM (Fig. 1e). We observed that both G9a and GLP SET-domains could methylate the N-terminus fragment of ATF7IP, whereas they could not when the K16 residue of ATF7IP was substituted with arginine (KR mutant). G9a and GLP SET-domains could also methylate full-length recombinant ATF7IP and the ATF7IP-family molecule ATF7IP2, which also contains the H3K9-like lysine that was included in our identified candidates (Additional files 1 and 3: Table S1 and Fig. S2). These results show that our screen successfully identified G9a and GLP targets containing the histone H3K9-mimic sequence.

### ATF7IP is tri-methylated by G9a/GLP in mESCs

**Fig. 2:**
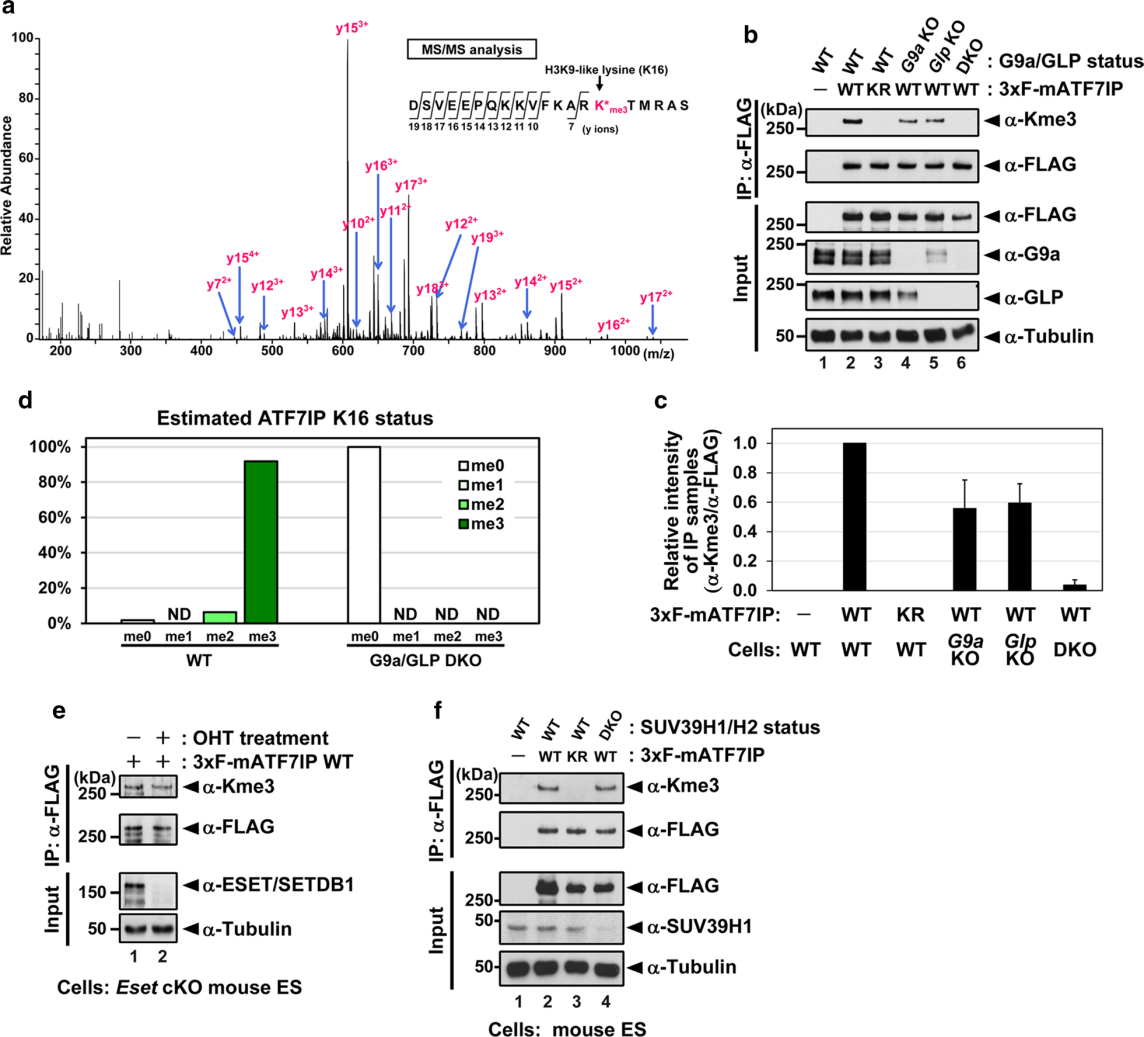
ATF7IP is tri-methylated by G9a/GLP in mESCs. **a**–**f** 3xFLAG-tagged ATF7IP was immunopurified from mESC lines and subjected to LC–MS/MS analysis or western blot analysis. **a** A tandem mass (MS/MS) spectrum of the tri-methylated “DSVEEPQKKVFKARKTMRAS” peptide from wild-type (WT) mESCs expressing 3xFLAG-ATF7IP. The detected y ions (pink) indicated tri-methylation occurred on the H3K9-like lysine (K16, pink). **b** tri-methylation status of exogenous ATF7IP in mESC lines mutant for *G9a*, *Glp,* or both. After an immunoprecipitation with FLAG M2 beads, western blot analysis was done with indicated antibodies. WT and KR indicate wild-type and K16R mutant of ATF7IP, respectively. **c** Quantification from the blots in (**b**) is shown. Error bars indicate ± SEM. **d** Percentage of methylation states of the ATF7IP K16 in WT or *G9a*/*Glp* DKO mESCs, as estimated by MS chromatogram. ND, not detected. **e** methylation status of exogenous ATF7IP was not affected by depletion of SETDB1/ESET. After serial treatment of tamoxifen (OHT), 3xFLAG-tagged ATF7IP was immunoprecipitated by FLAG M2 beads. Western blot analysis was done with indicated antibodies. Efficient knock-out in the tamoxifen-treated cell population was confirmed by the blot with anti-SETDB1 antibody. **f** Methylation status of exogenous ATF7IP in *Suv39h1*/*h2* DKO mESCs was comparable to that in WT cells

We then sought to examine the methylation status of ATF7IP in mESCs. For this, we established *Atf7ip* KO cells with the CRISPR-CAS9/gRNA gene KO method using mouse JM8 mESCs constitutively expressing Cas9 and integrating the murine stem cell virus (MSCV)-GFP reporter into the genome [34, 35]. After establishment of the *Atf7ip* KO line, either 3xFLAG-tagged ATF7IP WT or a K16R mutant was re-expressed in the KO cell line. The rescued ATF7IP showed protein expression levels similar to endogenous expression (Additional file 4: Fig. S3). We immunopurified exogenous 3xFLAG-ATF7IP with an anti-FLAG antibody and analyzed the methylation status of its H3K9-like lysine (K16) by mass spectrometry. We observed a tri-methylated form of the mouse ATF7IP (residues 2–21) peptide “DSVEEPQKKVFKARKTMRAS”, and MS/MS analysis confirmed that methylation occurred on K16 (Fig. 2a; a list of selected peaks is in Additional file 5: Table S2).

To examine whether G9a and GLP are required for tri-methylation of ATF7IP, we introduced 3xFLAG-ATF7IP into mESC lines deficient for *G9a*, *Glp,* or both and obtained a stably expressing cell line for each. After purification of 3xFLAG-ATF7IP from each cell line, the purified proteins were subjected to western blot analysis (Fig. 2b, quantification in Fig. 2c). The anti-FLAG antibody shows that purified ATF7IP occurred at comparable amounts in each group. Consistent with mass spectrometric analysis, the pan-tri-methylated lysine (Kme3) antibody shows a strong ATF7IP WT band but no ATF7IP K16R mutant band (Fig. 2b, c, lane 2 vs 3). Also, the anti-Kme3 antibody gave the strongest signal in WT cells, reduced signal in either *G9a* or *Glp* single KO cells, and no signal in the *G9a*/*Glp* DKO cells (Fig. 2b, c, lane 2, 4, 5, and 6). We then estimated the methylation status of ATF7IP K16 in the WT cells and the *G9a*/*Glp* DKO mESCs by immunoprecipitation of 3xFLAG-ATF7IP followed by mass spectrometric analysis. As shown in Fig. 2d, most ATF7IP (~ 92%) was tri-methylated in WT cells, whereas it was not methylated in the *G9a*/*Glp* DKO cells. We next investigated whether another H3K9 methyltransferase ESET/SETDB1, a binding partner of ATF7IP [27], or SUV39H1/H2 are involved in ATF7IP tri-methylation. To assess this question, we introduced 3xFLAG-ATF7IP into conditional KO mESCs for *Setdb1* [36] or *Suv39h1/h2* DKO mESCs [37]. We immunopurified 3xFLAG-ATF7IP from each KO cell and analyzed them by western blot (Fig. 2e, f). The Kme3 status of 3xFLAG-ATF7IP was not affected by the absence of SETDB1 or SUV39H1/H2. These data indicate that G9a and GLP are the enzymes responsible for ATF7IP K16 methylation in mESCs.

### G9a and GLP catalytic domains bind preferentially to unmethylated ATF7IP

**Fig. 3:**
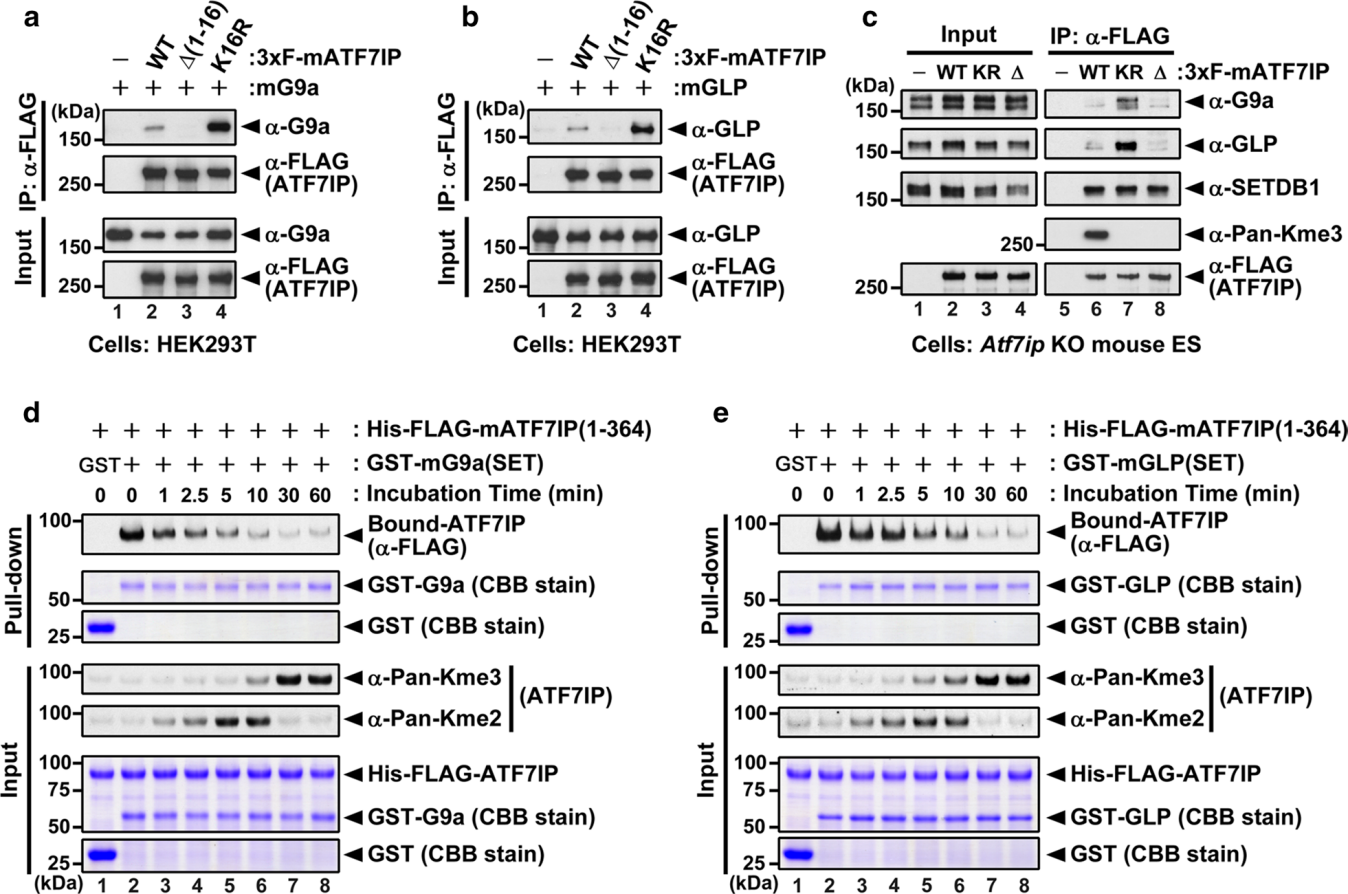
G9a/GLP binds preferentially to unmethylated ATF7IP and its K16R mutant. **a**, **b** Co-IP assay using anti-FLAG M2 beads and HEK293T cells co-transfected with G9a (**a**) or GLP (**b**) plus either 3xFLAG-tagged ATF7IP WT, Δ(1–16) mutant, or K16R mutant. **c** mESC lines expressing 3xFLAG-ATF7IP WT, K16R, or Δ(1–16) mutant were used for co-IP assay with anti-FLAG M2 beads. Co-IPed G9a, GLP, and SETDB1 were detected by western blot analysis. **d**, **e** In vitro methylation followed by in vitro binding assay. Recombinant proteins produced and purified from *E. coli* were incubated with SAM for the indicated times, and the reactants were then used for western blot analysis or GST-pull-down assay. Pulled-down ATF7IP (residues 1–364) recombinant protein was detected by western blot analysis. The interaction of ATF7IP with both G9a (**d**) and (**e**) was decreased in the incubation time-dependent manner

We then examined the interaction of ATF7IP with G9a and GLP. Either 3xFLAG-tagged ATF7IP WT, K16R mutants, or Δ(1–16) mutants lacking the 16 amino-acid N-terminus residues that were co-transfected with G9a (Fig. 3a) or GLP (Fig. 3b) into HEK293T cells. We performed co-immunoprecipitation (co-IP) assays using an anti-FLAG M2 agarose gel. As shown in Fig. 3a, b, both G9a and GLP could be co-immunoprecipitated (co-IPed) with WT ATF7IP but not with the Δ(1–16) mutant. Additionally, G9a and GLP were more abundantly co-IPed with K16R mutants than those co-IPed with WT ATF7IP. We then analyzed these interactions in *Atf7ip* KO mESCs rescued with 3xFLAG-ATF7IP WT or mutants. We again found that G9a and GLP were more efficiently co-IPed with the ATF7IP K16R mutant than with WT or Δ(1–16) mutant, whereas all the WT, K16R, and Δ(1–16) mutants showed comparable binding to SETDB1 (Fig. 3c). These data indicate that K16 of ATF7IP is responsible for these interactions and the methylation of ATF7IP K16 by G9a or GLP diminishes their binding affinities.

To further test this prediction, we performed an in vitro binding assay with recombinant proteins produced in and purified from bacteria. In this experiment, purified His and 3xFLAG-tagged ATF7IP (1–364) protein was incubated with either GST or GST-fused with the catalytic domain of G9a or GLP. We then added a methyl-cofactor, SAM, at several different time points and collected them simultaneously. Collected samples were then divided into two groups: one was used to examine the methylation status of each ATF7IP by western blot analysis, and the other was used to examine the amount of ATF7IP WT pulled-down by the GST-G9a or -GLP catalytic SET-domain by western blot analysis. These results showed that, after methyl-cofactor addition, ATF7IP was methylated by G9a or GLP in a time-dependent manner and the amount of ATF7IP pulled-down by the G9a or GLP SET-domains was inversely correlated with the incubation time of the cofactor addition and the methylation status of ATF7IP (Fig. 3d, e). Taken together, these results demonstrated that G9a and GLP catalytic domains bind to ATF7IP and they bind more strongly to the unmethylated form of ATF7IP than the methylated form. This property of the binding between ATF7IP and G9a/GLP fits the general concept of enzyme–substrate interaction.

### Binding affinities and kinetics of the G9a catalytic domain to its substrates

**Fig. 4:**
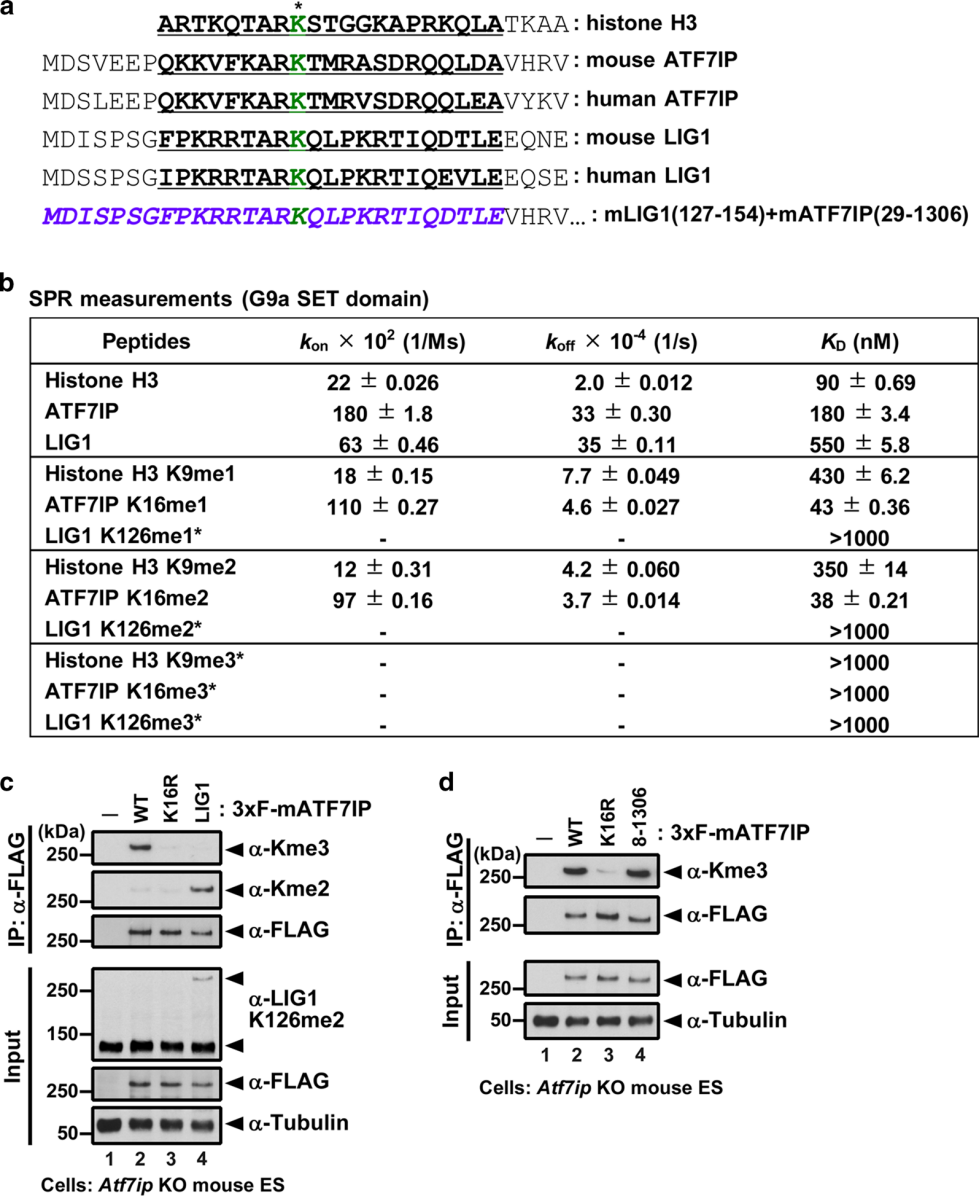
Interaction between the G9a SET-domain and peptides for histone H3, ATF7IP, or LIG1. **a** An alignment of the sequences for histone H3, mouse/human ATF7IP, mouse/human LIG1, and ATF7IP mutants. Underline indicates the sequence for the peptide used in this study. The methylated lysine was indicated in green. **b** SPR measurements of the interaction with the G9a SET-domain and peptides. Data are mean ± SE. *K*_D_ values were calculated from the *k*_on_ and *k*_off_ values when they are indicated. When kinetics were not fitted, *k*_on_ and *k*_off_ are shown in (–) and *K*_D_ values were calculated by steady-state affinity analysis. **c** 3xFLAG-tagged ATF7IP was immunopurified from mESC lines and subjected to western blot analysis. LIG1 (: 3xF-ATF7IP) indicates a chimeric protein composed of mLIG1 (residues 127–154) and ATF7IP (residues 29–1306). **d** 3xFLAG-tagged ATF7IP was immunopurified from mESC lines and subjected to western blot analysis. 8–1306 (: 3xF-ATF7IP) indicates a deletion mutant of ATF7IP (residues 8–1306)

We recently showed that the other G9a/GLP non-histone substrate LIG1 is mainly di-methylated in mESCs [19]. Since most of ATF7IP is tri-methylated in mESCs (Fig. 2d), we hypothesized that G9a/GLP may show a higher affinity for ATF7IP than LIG1. To examine binding affinity and kinetics, we used surface plasmon resonance (SPR) with the SET-domain of human G9a and either human ATF7IP, human LIG1, or histone H3 peptides containing a C-terminus biotin and unmodified or mono-, di-, or tri-methylated lysine at the H3K9 position (Fig. 4a). Peptides were immobilized on a streptavidin-coated sensor chip, and various concentrations of the G9a SET-domain were injected onto the sensor. We observed the interaction of the G9a SET-domain with all the peptides in a concentration-dependent manner.

The fitting of sensorgrams to a 1:1 binding model revealed the kinetic parameters for peptide-G9a interactions (Fig. 4b; a representative sensorgram is shown in Additional file 6: Fig. S4). In the unmodified state, the *k*_off_ value of H3 (2.0 × 10^−4^ s^−1^) was ~ 17-fold slower than those of ATF7IP (33 × 10^−4^ s^−1^) and LIG1 (35 × 10^−4^ s^−1^). In the mono- and di-methylated status, the *k*_off_ values of H3 became twofold–fourfold faster than that of the unmodified status (7.7 × 10^−4^ s^−1^ for K9me1 and 4.2 × 10^−4^ s^−1^ for K9me2), resulting in an increase in the dissociation constants (*K*_D_) by methylation (90 nM for K9me0, 430 nM for K9me1, and 350 nM for K9me2). Interestingly, the *k*_off_ values of ATF7IP became sevenfold to ninefold slower by methylation (4.6 × 10^−4^ s^−1^ for K16me1 and 3.7 × 10^−4^ s^−1^ for K16me2), resulting in a decrease in *K*_D_ (180 nM for K16me0, 43 nM for K16me1, and 38 nM for K16me2). On the other hand, we could not obtain kinetic parameters for LIG1 K126me1 and me2 peptides due to too rapid dissociation patterns. In the tri-methylated state, now kinetic parameters for none of the analyzed peptides were obtained; we therefore determined, by steady-state affinity analysis, that all of these *K*_D_ values were over 1000 nM (Fig. 4b). These data indicate that mono- or di-methylated ATF7IP are better substrates for G9a than unmodified ones, whereas unmodified H3 or LIG1 is better than mono- or di-methylated ones, respectively.

### Methylation status of LIG1-ATF7IP chimeric protein in mESCs

By SPR analyses, we saw a large difference in the affinities of di-methylation between ATF7IP and LIG1, which may provide an explanation for the different methylation status of ATF7IP compared to that of LIG1 in mESCs. To test whether the N-terminus region of ATF7IP determines its methylation status, we used the expression vector for LIG1-ATF7IP chimeric proteins composed of mLIG1 (residues 127–154) and ATF7IP (residues 29–1306) (Fig. 4a). After FLAG-IP from mESCs expressing 3xFLAG-ATF7IP WT or the chimeric protein, we examined the methylation status by western blot analysis (Fig. 4c). The anti-Kme3 antibody detected an ATF7IP band but not a LIG1-ATF7IP chimeric protein or ATF7IP K16R mutant. In contrast, the anti-Kme2 antibody shows a strong LIG1-ATF7IP chimeric protein band, while it shows no ATF7IP WT or ATF7IP K16R mutant bands. In addition, we also detected a band corresponding to the LIG1-ATF7IP chimeric protein molecular weight with an anti-LIG1 K126me2-specific antibody [19]. Furthermore, we found that the deletion of the first seven residues of ATF7IP does not affect the tri-methylation status in mESCs (Fig. 4d). These data show that the LIG1ATF7IP chimeric protein is subjected to di-methylation rather than tri-methylation and suggest that the ATF7IP N-terminus region (residues 8–28) determines its methylation status by the G9a/GLP complex in cells.

### Identification of MPP8 as a read out for methylated ATF7IP

**Fig. 5:**
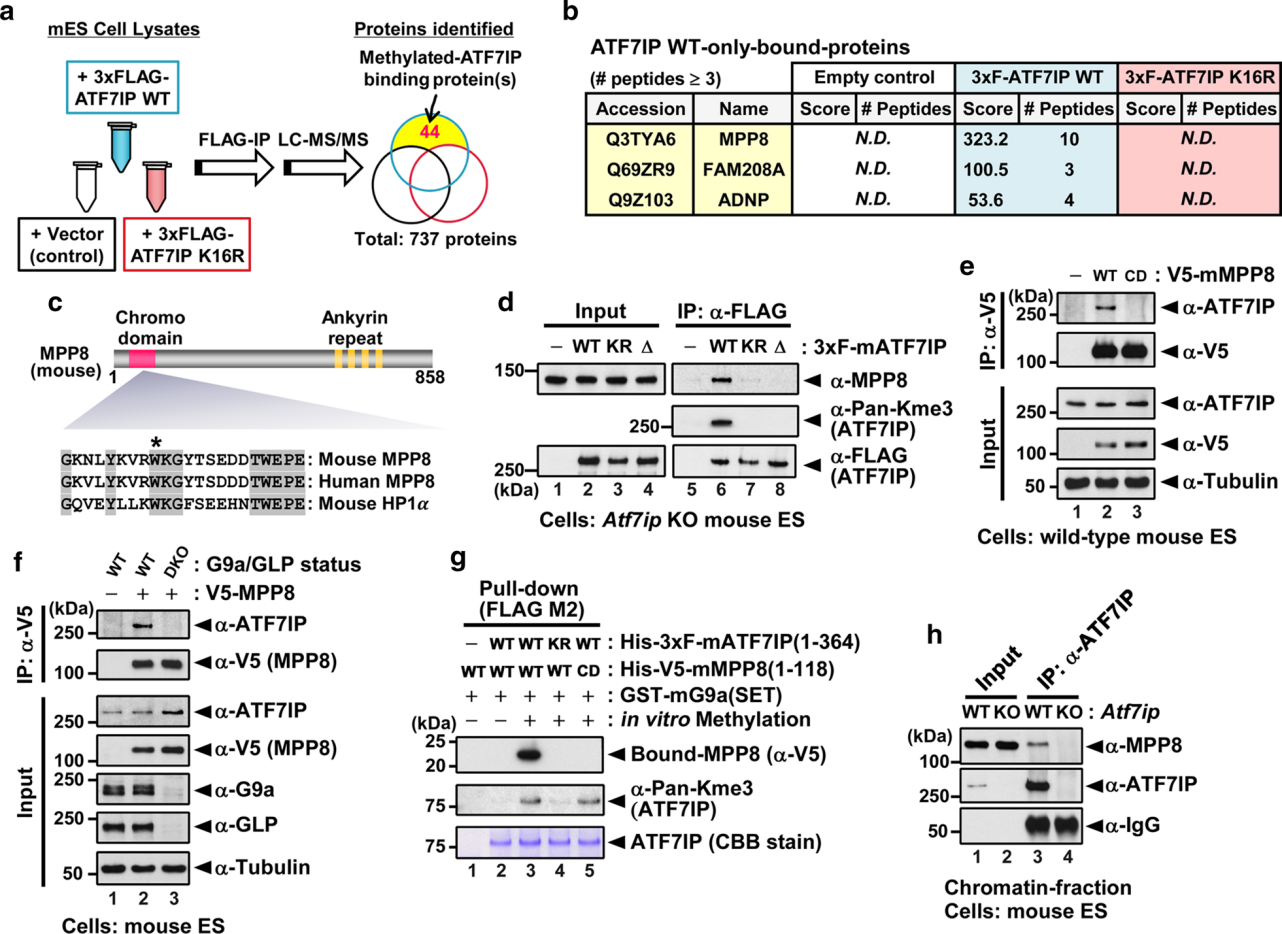
The MPP8 chromodomain recognizes methylated ATF7IP. **a** A scheme for the identification of the proteins bound to ATF7IP WT or K16R mutant. The three cell lines expressing 3xFLAG-ATF7IP, K16R mutant or nothing were used for the immunoprecipitation with anti-FLAG M2 beads followed by LC–MS/MS analysis. A Venn diagram shows the number of bindings proteins specifically for ATF7IP WT. **b** Top three proteins identified as ATF7IP WT-only-bound proteins. **c** A schematic of mouse MPP8 protein. Known domains and an alignment of mouse and human MPP8 and mouse HP1a are shown. An asterisk indicates a key residue (W80) for the interaction with H3K9me3. **d** Co-IP assay using *Atf7ip* KO mESCs rescued by ATF7IP WT, K16R, or Δ(1–16) mutant. Bound endogenous MPP8 was detected by western blot analysis. **e** The interaction of MPP8 with ATF7IP via its chromodomain. Co-IP assay with anti-V5 antibody was performed using WT mESCs expressing V5-MPP8 WT or W80A mutant (CD, chromodomain dead). Bound endogenous ATF7IP was detected by western blot analysis. **f** The interaction of MPP8 with ATF7IP requires G9a and GLP. Co-IP assay with anti-V5 antibody was performed using WT or *G9a*/*Glp* DKO mESCs expressing V5-MPP8 WT. Bound endogenous ATF7IP was detected by western blot analysis. **g** In vitro methylation followed by in vitro binding assay with anti-FLAG. Recombinant proteins were produced in and purified from *E. coli*. The interaction of MPP8 with ATF7IP was detected by western blot analysis. KR, K16 R mutant. CD, chromodomain dead (W80A mutant). **h** Co-IP assay with anti-ATF7IP antibody using a chromatin fraction from WT or *Atf7ip* KO mESCs. The endogenous interaction of MPP8 with ATF7IP was detected by western blot analysis

**Fig. 6:**
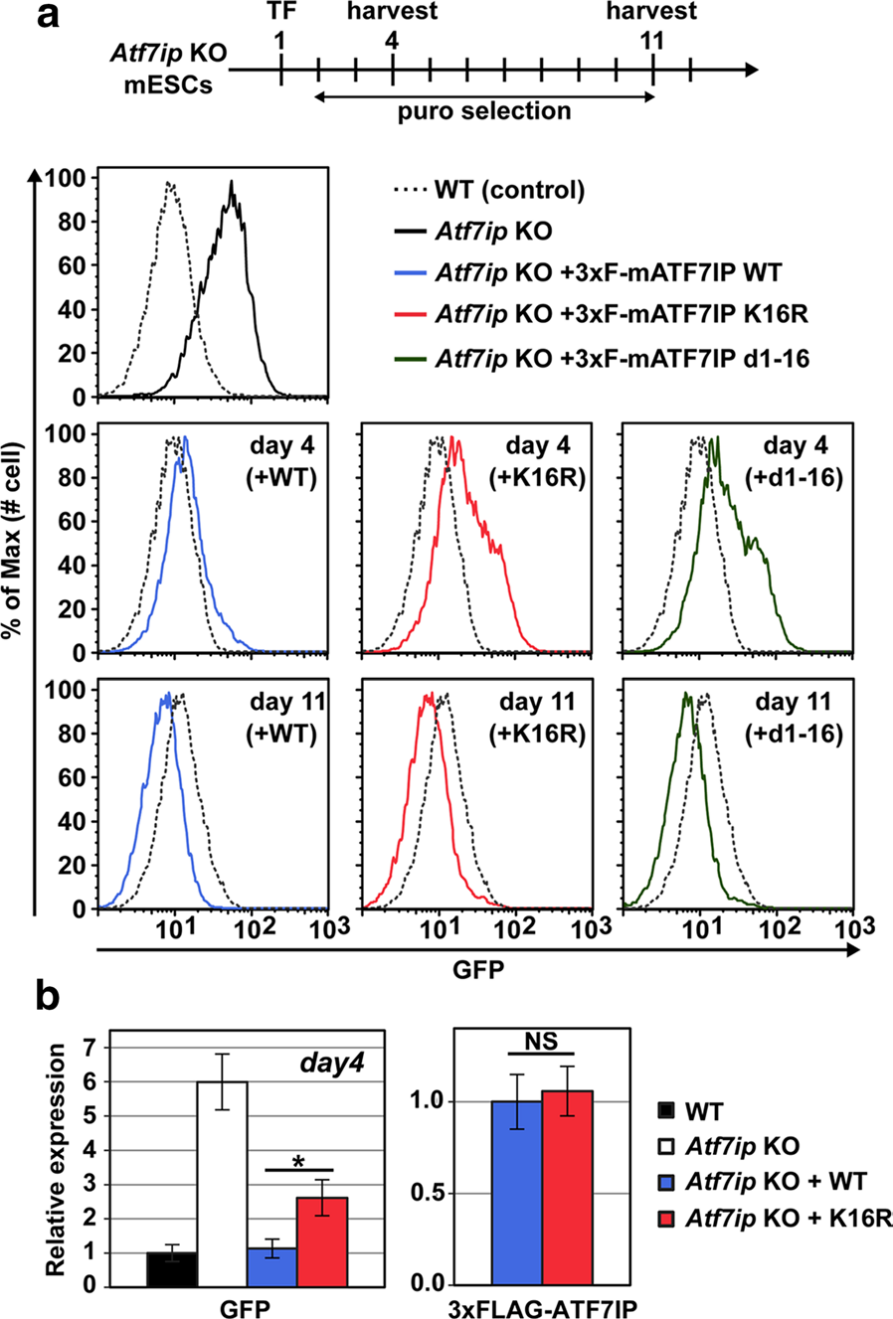
Methylation of ATF7IP plays a role in the silencing transgene integrated by retrovirus vector in mESCs. **a** MSCV-GFP expression was analyzed by flow cytometric analysis at indicated time points. The transfected cells were subjected to continuous drug selection from day 2 after the transfection. **b** RT-qPCR analysis was performed with samples collected at day 4 after the transfection. RNA expression was normalized to *Hprt* expression and is shown relative to the level in WT cells without the case of 3xFLAG-ATF7IP. For 3xFLAG-ATF7IP mRNA, RNA expression is shown relative to the level in the ATF7IP WT-rescued cells. Data are mean ± SEM; *n* = 4, biological replicates. NS: *P* > 0.05, *P < 0.05 by unpaired Student’s *t* test

We next sought to identify binding protein(s) specific to the methylated form of ATF7IP. To this end, we performed immunoprecipitation with an anti-FLAG antibody using cell lysates from *Atf7ip* KO mESCs expressing either 3xFLAG-ATF7IP WT, mutant K16R, or neither (see schematic in Fig. 5a). We analyzed the immunoprecipitates by LC–MS/MS and identified 44 proteins detected only in the immunoprecipitates of WT ATF7IP (Fig. 5a and Additional file 7: Table S3). We found MPP8, FAM208A (also known as TASOR), and ADNP as the top three proteins of ATF7IP WT-only binding proteins, suggesting that these proteins may bind specifically to methylated ATF7IP (Fig. 6b). We focused on MPP8 because MPP8 is known to bind to H3K9me3 and di-methylated DNMT3a that is catalyzed by G9a/GLP (Fig. 5c) [38–40]. We performed IP followed by western blot analysis using *Atf7ip* KO mESCs expressing 3xF-ATF7IP WT, K16R, or Δ(1–16) mutants and confirmed that ATF7IP WT, but not K16R nor Δ(1–16) mutants, co-IPed endogenous mouse MPP8 (Fig. 5d). MPP8 recognized H3K9me3 via its chromodomain (CD), and the substitution of tryptophan 80 to alanine (W80A) hindered the interaction with H3K9me3 (Fig. 5c) [38, 39]. We tested whether the CD of MPP8 is also required for its interaction with methylated ATF7IP by co-IP assay using mESCs expressing V5-MPP8 WT or the W80A mutant (Fig. 5e). We found that MPP8 WT, but not the W80A mutant, co-IPed endogenous ATF7IP, suggesting the H3K9me3 binding of MPP8 CD is essential for its recognition of methylated ATF7IP. We then examined whether ATF7IP methylation by G9a/GLP is required for ATF7IP interaction with MPP8. To this end, we performed a co-IP assay using WT or *G9a*/*Glp* DKO mESCs expressing V5-MPP8 WT. As shown in Fig. 5f, MPP8 co-IPed endogenous ATF7IP in WT cells but not in *G9a*/*Glp* DKO cells. To further confirm methylation-dependent binding, we performed an in vitro binding assay combined with in vitro methylation using purified recombinant proteins (Fig. 5g). This result shows that the interaction of MPP8 with ATF7IP in vitro required the methylation of ATF7IP at K16 and the CD of MPP8, indicating MPP8 binds specifically to ATF7IP methylated at K16 via its CD. Taking all the above into consideration, we propose that MPP8 functions not only as a reader for methylated H3K9, but also as a reader for methylated ATF7IP. We further examined the endogenous interaction of ATF7IP with MPP8 using the chromatin fraction of mESCs (Fig. 5h). These results showed that ATF7IP could bind to MPP8 at the endogenous level and suggest that the interaction could occur in chromatin.

### ATF7IP methylation plays a role in transgene silencing integrated by a retrovirus vector in mESCs

We then sought to identify the role(s) of ATF7IP methylation and its recognition by MPP8. Recent studies identified MPP8 as a component of the human silencing hub (HUSH) complex, which is recruited to the transgene-carrying retrovirus integrated into heterochromatin and silences transgene expression by recruiting SETDB1 in human cells [41]. Also, it has been reported that ATF7IP plays a crucial role in transgene silencing by the HUSH complex and SETDB1 [31]. We therefore asked whether ATF7IP methylation at the histone-mimic is involved in HUSH/SETDB1-mediated transcriptional silencing of the transgene. Since we, and others, previously showed that both SETDB1 and ATF7IP silence the GFP-carrying MSCV retrovirus in mESCs [29, 35, 36], we used *Atf7ip* KO mESCs infected with the MSCV-GFP retrovirus reporter. As expected, we observed that the established *Atf7ip* KO in mESCs increases GFP expression compared to the parental WT cells at the mRNA and protein levels (Fig. 6a, b). In addition, *Mpp8* KO mESCs also showed increased GFP expression (Additional file 8: Fig. S5A–C). We transfected 3xFLAG-tagged ATF7IP with either WT, K16R, or Δ(1–16) mutants into *Atf7ip* KO mESCs and selected transfected cells by continuous drug (puromycin) treatment. Then, we monitored the GFP reporter expression at days 4 and 11, after transfection, by flow cytometric analysis (Fig. 6a). We observed that GFP transgene expression was shut-down by WT ATF7IP by day 4 but part of the population still expressed a high level of GFP in the cells transfected with K16R and the Δ(1–16) mutant ATF7IP. On day 11 post-transfection, the GFP transgene was also completely silenced in cells expressing such mutants. We also performed reverse transcription-quantitative PCR (RT-qPCR) analysis and again observed the increased GFP mRNA expression in K16R mutant-rescued cells, compared to the WT-rescued cells at day 4 (Fig. 6b, left). We confirmed that there is comparable exogenous *Atf7ip* mRNA expression between the rescued cell lines (Fig. 6b, right). These data suggest that K-methylation inactive ATF7IP mutants are still capable of SETDB1-mediated gene silencing but its function is partially impaired, implying a potential role of ATF7IP methylation-dependent binding to the HUSH complex subunit MPP8 in the HUSH/SETDB1-mediated transgene silencing.

## Discussion

We, and others, have previously performed a substrate screen of G9a/GLP and found some potential methylation substrates [16, 42]. However, recent work found previously unidentified non-histone substrates of G9a and GLP and their biological functions [17–19]. These results suggest that the biological roles of G9 and GLP are mediated, in part, through the methylation of non-histone substrates. Since it is still possible that more physiological substrate(s) of G9a/GLP exist, we here performed an additional unbiased substrate candidate screen using a chemical tag (ProSeAM), exogenous recombinant catalytic domain of G9a or GLP, and lysates from *G9a*/*Glp* DKO mESCs where endogenous methylations by them should be completely diminished (Fig. 1). This strategy could enhance detection of endogenous substrates with a high S/N ratio. By this screen, we identified 59 proteins, including histones and other known G9a/GLP substrates containing H3K9-like sequences [16, 19] and not-reported ones. Among the 59 proteins, 7 and 55 were G9a and GLP target candidates, respectively (three of them overlapped). Currently, we do not know why the GLP SET-domain screen identified more candidates than G9a SET-domain screen. It has been reported that GLP shows higher catalytic activity with ProSeAM than G9a [22]. This might create such differences.

We show here that ATF7IP is primarily tri-methylated in mESCs (Fig. 2). This is not the case for other G9a/GLP substrates, such as histone H3 and LIG1 in vivo. G9a/GLP are capable of tri-methylating H3 and LIG1 under in vitro conditions [19, 43], suggesting that there may be a regulation mechanism that restricts LIG1 tri-methylation or allows tri-methylation of ATF7IP in cells. Based on our observation that the affinity of ATF7IP K16me2 for the SET-domain of G9a was much higher than that of LIG1 K126me2 (Fig. 4b), we suggest that G9a/GLP could methylate the di-methylated ATF7IP, but not the di-methylated LIG1, to create a tri-methylated form under physiological conditions. As for histone H3, we observed that G9a shows similar affinity to ATF7IP. This suggests that histone H3 could be tri-methylated more easily than LIG1. However, genetic studies show that G9a/GLP is mainly responsible for di-methylation of H3K9 in cells and mice [11]. This could be explained by a potential mechanism that restricts G9a/GLP-mediated H3K9 methylation to a di-methylation or by a potential trimming mechanism that requires a histone H3K9 de-methylase. Further studies will be required to address this issue.

It is unclear whether tri-methylation of ATF7IP in mESCs is only accomplished by the G9a/GLP complex. Our genetic study shows that methylation of ATF7IP is only inhibited in the absence of both G9a and GLP, while depletion of other major H3K9 methyltransferase SUV39H1/H2 or SETDB1 did not affect methylation. This highly suggests that G9a and GLP are redundantly important and mostly sufficient for the K16 tri-methylation of ATF7IP.

**Fig. 7:**
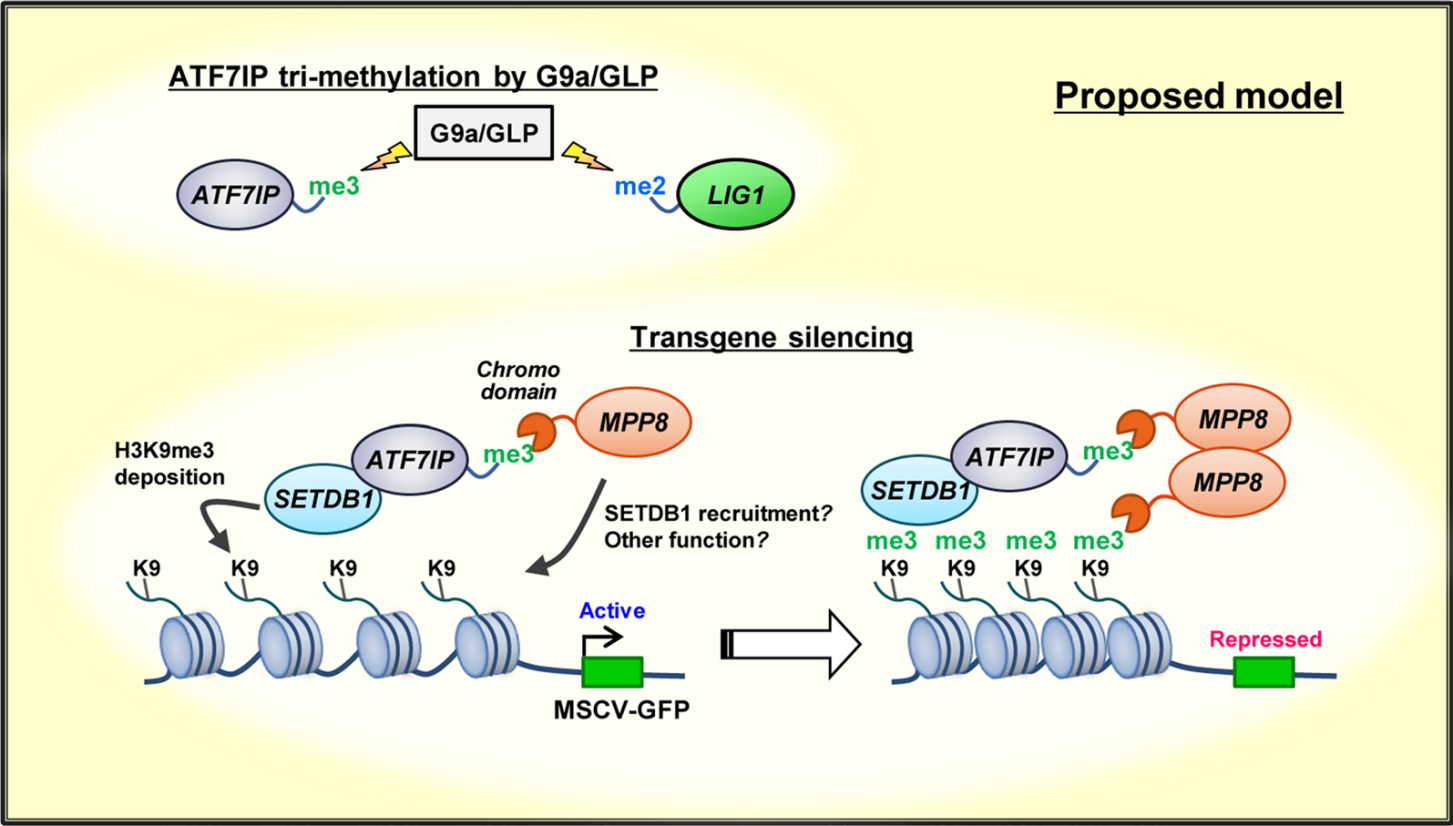
Proposed model of ATF7IP methylation and its potential function

As ATF7IP is bound to MPP8 in the chromatin fraction (Fig. 5h), we expect that the ATF7IP-MPP8 complex may function in gene regulation. Emerging reports reveal the HUSH complex, comprised of MPP8, FAM208A (also known as TASOR), and Periphilin, mediates silencing of a transgene integrated into heterochromatin by recruiting SETDB1 and MORC2 [41, 44, 45]. In addition, ATF7IP was also shown to be involved in the HUSH-mediated heterochromatin formation [31]. We found that methylated ATF7IP is bound by the HUSH subunit MPP8 via the chromodomain of MPP8 and that another HUSH subunit, FAM208a, was a second-ranked hit in a screen of binding proteins for methylated ATF7IP (Fig. 6 and Additional file 7: Table S3). We speculate that FAM208A enrichment in this assay is probably indirect and mediated by MPP8, because MPP8 and FAM208A form a tight complex [41]. Based on these data, we hypothesize that the methylation-dependent complex formation of ATF7IP with MPP8 is involved in HUSH/SETDB1-mediated heterochromatin formation at a transgene (Fig. 7). Indeed, we show that the methylated lysine residue of ATF7IP is required for efficient silencing of the GFP transgene reporter in mESCs (Fig. 6).

## Conclusions

Our screen succeeded in the identification of known and previously unknown G9a/GLP targets in mESCs. Of the novel substrate candidates, we focused on ATF7IP and revealed (1) its unexpected tri-methylation by G9a and GLP, (2) methylation-dependent complex formation between ATF7IP and MPP8, and (3) potential role of ATF7IP lysine methylation in SETDB1/HUSH-mediated transgene silencing. Future studies should clarify a detailed mechanism for the function of a methylated ATF7IP-MPP8 complex formation in transgene silencing.

## Methods

### Cell culture and DNA transfection

mESCs were cultured in Dulbecco’s modified Eagle’s medium (DMEM, Cat# D6429; Sigma-Aldrich, USA) supplemented with 15% KnockOut Serum Replacement (KSR; Cat# 10828028, Invitrogen, USA), 1% fetal bovine serum, 0.1 mM β-mercaptoethanol, leukemia-inhibiting factor, and 1 × non-essential amino acids. HEK293T cells were cultured in DMEM (Cat# 08457-55; Nacalai Tesque, Japan) supplemented with 10% FBS. The mESCs lines used in this study are as follows: TT2 wild-type and its derivatives, *G9a* KO; clone name: 2-3), *Glp* KO (clone name: CD12), and *G9a*/*Glp* DKO (clone name: 248-6) [9, 11]; JM8 constitutively expressing hCas9 infected with murine stem cell virus-GFP (MSCV-GFP) (clone name: #7) [34, 35] and its derivatives, *Atf7ip* KO (clone name: TT#2-5 and TT#2-12) and *Mpp8* KO (clone name: #1 and #2); TT2 *Setdb1* conditional KO [36]; *Suv39h1*/*h2* DKO mESCs [37]. *Atf7ip* KO and *Mpp8* KO cells were established by CRISPR/Cas9 technology using pKLV2-U6gRNA5(Bbs1)-PGKpuro2ABFP [46]. Target sequneces of gRNAs are listed in Additional file 9: Table S4. The clone TT#2-12 was mainly used in this study, whereas the clone TT#2-5 was used for the validation of the results. Stably transfected cell lines were established by the piggyBac transposon system. DNA transfection was performed using Lipofectamine 2000 (Invitrogen, USA; for mESCs) or Polyethylenimine Max (Polyscience, Inc., USA; for HEK293T cells), according to the manufacturer’s instructions.

### Plasmids

pGEX vectors for production of GST-fusion with mouse G9a SET-domain and mouse GLP SET-domain have been described previously [8, 26]. pCR2.1 vector for production of human G9a SET-domain with an in vitro translation system has been described previously [47]. Briefly, the DNA fragment containing the transcription promoter sequence of T7 RNA polymerase, the sequence encoding an N-terminal histidine-rich affinity tag (N11-tag), cDNA encoding human G9a (residues 913–1193), a stop codon, and the T7 transcription terminator sequence were inserted into pCR2.1. The human G9a protein was designed to have N11-tag followed by a TEV-protease recognition sequence. Full-length mouse *Atf7ip* cDNA was generated from FANTOM clones AK167542 and AK157479. Mouse *Atf7ip* fragments were inserted into pET-19b vector for production of recombinant proteins in *E. coli*. pPiggyBack-CAG-IRES-puromycin vectors expressing ATF7IP and mouse MPP8 were generated by In-Fusion cloning. Substitution or deletion of residues was performed by site-directed mutagenesis or over-lapping PCR. The detailed information of plasmids and used primers is given in Additional file 9: Table S4.

### Antibodies

The following antibodies are used for this study: anti-α-Tubulin (clone B-5-1-2, Sigma-Aldrich); anti-FLAG M2 antibody (F3165, Sigma-Aldrich); anti-V5 antibody (R960-25, Thermo Fisher which is same as #46-0705, Life technology); anti-G9a (#8620,(11)); anti-GLP (#0422,(11)); anti-SUV39H1 (clone D11B6, Cell signalling); anti-MCAF1/ATF7IP [48] and anti-ATF7IP (ab84497, Abcam); anti-MPP8 (16796-1-AP, Proteintech); anti-H3 (07-690, Millipore); anti-Pan-Kme2 (c4930/c5123 mix.(19)); anti-Pan-Kme3 (76118, Abcam); anti-SETDB1/ESET (cp10377, Cell Applications).

### Purification of recombinant proteins

*Escherichia coli* (*E. coli*) BL21 (pLysS) strains were transformed with expression vectors, and the transformed bacteria were cultured in 2× YT medium with antibiotics and 500 µM isopropyl β-D-1-thiogalactopyranoside (IPTG) for 16 h at 16 °C. His-tagged proteins were purified as described previously [19]. For purification of GST-fusion proteins, the cultured cell pellets were lysed with lysis buffer (1xPBS/0.5% NP-40) added with phenylmethanesulfonyl fluoride (PMSF) at 1 mM. After sonication with a Branson Sonifier (S-250D, Branson Ultrasonics) for 10 or 20 min on ice, the lysates were centrifuged at 7300×*g* for 10 min. The supernatants were incubated with pre-washed glutathione 4B Sepharose (GE) for 1–3 h at 4 °C with gentle rotation. The beads were washed 5 times with the lysis buffer and then eluted with elution buffer [50 mM Tris, 10 mM glutathione, 1 mM dithiothreitol (DTT)]. The eluted proteins were dialyzed with 1xPBS/10% glycerol, and the concentration was measured by the Bradford protein assay.

For SPR measurements, human G9a SET-domain (residues 913–1193) was synthesized with an in vitro translation system, essentially as previously described [47, 49]. The synthesized protein was clarified by centrifugation. The supernatant was loaded onto a HisTrap HP column (GE Healthcare), and eluted with 20 mM Tris-HCl buffer (pH 8.0) containing 500 mM NaCl and 500 mM imidazole. The N-terminal histidine tag of human G9a protein was cleaved by incubating with TEV protease for 2 h at 25 °C. The tag-cleaved human G9a protein was further purified by anion exchange chromatography, followed by size-exclusion column chromatography using a HiLoad Superdex 200 16/60 (GE Healthcare) equilibrated with 10 mM HEPES buffer (pH 7.4) containing 150 mM NaCl.

### In vitro labelling of cell lysates with ProSeAM

Synthesis of ProSeAM was performed as previously described [25]. *G9a*/*Glp* DKO mESCs were cultured with SILAC medium over 2 weeks. The cells were lysed with lysis buffer (50 mM Tris-HCl at pH 8.0, 50 mM KCl, 10% glycerol, 0.1% Tween20). One hundred and twenty µg of the cell lysates were incubated with reaction buffer (50 mM Tris-HCl at pH 8.0) containing 400 µM ProSeAM and 6 µg recombinant GST-fusion with SET-domain of mouse G9a or mouse GLP for 2 h at 20 °C. The following click reaction, purification, and mass spectrometry were performed as previously described [25, 50].

### In vitro methylation assay

In vitro methylation assay with radioisotope was performed as previously described (Ferry et al. [19], Mol Cell). Briefly, one microgram of recombinant His-ATF7IP proteins were incubated in reaction buffer (50 mM Tris-HCl pH 8.0) with either 1 µg of GST, GST-G9a SET or -GLP SET and ^14^C-labelled SAM (0.01 mCi, Perkin Elmer) for 2 h at 30 °C. The reaction was stopped by adding Laemmli SDS sample buffer. Proteins were resolved on acrylamide SDS-PAGE gel, the dry gel was exposed to an imaging plate (FUJI-FILM) for 48 h, and the autoradiographic signal was detected using a BAS-5000 Image analyzer (FUJI-FILM).

### Western blot and quantitative PCR analysis

For western blot analysis, cell lysates were prepared as follows: the cells were harvested after trypsinization, washed once with ice-cold PBS, and lysed with high-salt lysis buffer (50 mM Tris-HCl at pH 7.5, 500 mM NaCl, 1 mM EDTA, 10% glycerol, 1% Nonidet P-40, 1 mM PMSF, 1× protease inhibitor cocktail; Nacalai Tesque, Japan). The cell lysates were subjected to Bradford protein assay (Bio-Rad). Equivalent amounts of protein were resolved by SDS-PAGE and subjected to standard western blot using nitrocellulose membrane. The antibodies used in this study are described in “Antibodies” section. For quantitative PCR analysis, total RNA was isolated using RNeasy mini kit (Qiagen, Germany), including DNase treatment procedure on the column, according to the manufactures protocol. cDNAs were generated using Omniscript kit (Qiagen, Germany). Quantitative PCR analysis was performed using Power SYBR and Step-One Plus (Applied Biosystems). The primers used in these analyses are provided in Additional file 9: Table S4.

### Immunoprecipitation

For immunoprecipitation (IP), cells were lysed with normal-lysis buffer (50 mM Tris-HCl at pH 7.5, 150 mM NaCl, 1 mM EDTA, 10% glycerol, 1% Nonidet P-40, 1 mM PMSF, 1× protease inhibitor cocktail), mid-lysis buffer (50 mM Tris-HCl at pH 7.5, 300 mM NaCl, 1 mM EDTA, 10% glycerol, 1% Nonidet P-40, 1 mM phenylmethanesulfonyl fluoride (PMSF), 1× protease inhibitor cocktail; Nacalai Tesque, Japan) or the high-salt lysis buffer (see above). After centrifugation at 14,000×*g* for 10 min, the supernatants were incubated with anti-FLAG affinity gel (Sigma-Aldrich, USA) or antibody-conjugated Protein A and Protein G Dynabeads mix for at least 3 h to overnight at 4 °C. The resin was then washed five times with the lysis buffer and eluted by 2× Laemmli sample buffer. The eluate was resolved by SDS-PAGE, and gel staining was performed using CBB stain one (Nacalai). Equivalent amounts of the input and the precipitates were subjected to standard western blot analysis.

For immunoprecipitation using chromatin fraction, 6.0 × 10^7^ cells per sample were lysed with 4 mL Buffer A (10 mM HEPES at pH 7.9, 10 mM KCl, 1.5 mM MgCl_2_, 340 mM sucrose, 10% glycerol, 1 mM DTT, 1 mM PMSF, 1× protease inhibitor) containing Triton-X 100 at 0.1% as a final concentration and incubated on ice for 5 min. After centrifugation at 1300×*g* for 4 min at 4 °C, the pellet was re-suspended in 4 mL Buffer B (3 mM EDTA, 0.2 mM EGTA, 1 mM DTT, 1 mM PMSF, 1× protease inhibitor) and incubated on ice for 10 min. After centrifugation at 1700×*g* for 4 min at 4 °C, the pellet (chromatin fraction) was re-suspended in 1 mL normal-lysis buffer and sonicated. After centrifugation at 14,000×*g* for 10 min, the supernatants were incubated with antibody for overnight at 4 °C and were further incubated with Protein A and Protein G Dynabeads mix for at least 1 h at 4 °C. The resin was then washed five times with the lysis buffer and eluted by 2× Laemmli sample buffer.

### Mass spectrometry analysis for detection of ATF7IP K16 methylation

3xFLAG-ATF7IP was immunoprecipitated by FLAG M2 affinity gel, as described in “Immunoprecipitation” section. The immunoprecipitates were resolved by SDS-PAGE, and the protein bands of 3xF-ATF7IP were cut out and digested with an endoproteinase AspN (Roche Diagnostics International Ltd) in gel. The digests were applied to nano-liquid chromatography–tandem mass spectrometry using a Q Exactive mass spectrometer (Thermo Fisher Scientific). The peptide mixtures (2 μL) were separated by nano-ESI spray column (75 μm [ID] × 100 mm [L], NTCC analytical column C18, 3 μm, Nikkyo Technos) with a linear gradient of 0–35% buffer B (acetonitrile with 0.1% (v/v) formic acid) in buffer A (MilliQ water with 0.1% (v/v) formic acid) at a flow rate of 300 nL/min over 10 min (EAST-nLC 1000; Thermo Fisher Scientific). The mass spectrometer was operated in the positive-ion mode, and the MS and MS/MS spectra were acquired using a data-dependent TOP10 method. The MS/MS spectra and the MS chromatograms of fifth-charged none, mono-, di-, and tri-methylated “DSVEEPQKKVFKARKTMRAS” peptide ions were drawn using Qual Browser, Thermo Xcalibur 3.1.66.10 and each peptide amount was estimated by the peak area.

### Pull-down assay combined with in vitro methylation

Four microgram recombinant proteins were mixed in 20 µL of the reaction buffer (50 mM Tris-HCl pH 8.0) and pre-incubated at 30 °C. To start reaction, cold SAM (1 µL; stock concentration: 4 mM) was added at several time points and further incubated at 30 °C. The reactants were immediately transferred on ice and divided into two tubes. One was used for western blot analysis for the methylation status, and the other one was used for following pull-down assay. For pull-down assay, the half of reactants (10 µL) were diluted with 400 µL of ice-cold GST-binding buffer (20 mM Tris-HCl at pH 7.5, 50 mM NaCl, 1 mM EDTA, 10% glycerol, 0.5% NP-40, 1 mM PMSF, 1× protease inhibitor cocktail; Nacalai Tesque, Japan) and incubated with 100 µL GST-binding buffer containing Glutathione 4B Sepharose (20 µL as beads volume) at 4 °C for 4 h. After washing with the GST-binding buffer four times, the precipitates were eluted by 2× Laemmli sample buffer. The eluates were subjected to standard western blot analysis or CBB staining.

### Surface plasmon resonance (SPR) measurements

The SPR binding assay for human G9a SET-domain (residues 913–1193) was performed at 25 °C on a Biacore T200 instrument (GE Healthcare). Biotinylated peptides were immobilized on a streptavidin-coated Biacore sensor chip SA (GE Healthcare). The flow cell was blocked with the biotin labelling reagent EZ-Link Amine-PEG2-Biotin (Thermo Fisher Scientific). The purified human G9a SET-domain at concentrations from 20 μM or 10 μM were prepared by 1.5-fold serial dilution in running buffer (10 mM HEPES at pH 7.4, 150 mM NaCl). In the case of measurements of all tri-methylated peptides and LIG1 K126me2 peptide, human G9a SET-domain was prepared with a twofold dilution series from 200 μM. Association and dissociation times were 120 and 600 s at the flow rate of 30 μL/min, respectively. After each measurement, the surface was regenerated by injecting 50 mM NaOH and 1 M NaCl for 2 min. The sensorgrams were analyzed using BIAevaluation software (GE Healthcare). The response curves of various protein concentrations were fitted according to the 1:1 binding model.

### Mass spectrometry analysis for identification of ATF7IP binding proteins

mESCs cultured in a 15-cm dish were collected, lysed in normal-lysis buffer (see above), and sonicated on ice. After centrifugation at 14,000×*g* for 30 min, the supernatants were incubated with 5 µg anti-FLAG M2 antibody for overnight at 4 °C and were further incubated with 100 µL Protein G Dynabeads (30 mg/mL) for 1 h at room temperature. The resin was then washed six times with the lysis buffer and washed two times additionally with 100 mM ammonium bicarbonate (ABC) buffer. The pellet was re-suspended in 100 µL ABC buffer with 1/10 volume of acetonitrile and DTT (20 mM). The mixture was incubated at 56 °C for 30 min, and then, 6 µL of 500 mM iodoacetoamide was added and incubated at 37 °C for 30 min in the dark. The proteins were digested with 3 µg Trypsin/Lys-C mix (Promega) at 37 °C overnight. The digested protein fragments were applied to quantitative MS/MS analysis and following protein identification as described previously [25].

## Additional files

**Additional file 1: Table S1.** List of ProSeAM-based screen.

**Additional file 2: Fig. S1.** An *in vitro* methylation assay with 3xFLAG-tagged hNup107 protein, GST-tagged SET-domain of G9a or GLP, and ^14^C-labelled SAM. 3xFLAG-tagged hNup107 was expressed in and purified from HEK293 cells transfected with the expression vector. An incorporation of the methyl-moiety was detected as an autoradiographic signal. WT and KR indicate wild-type and K25R mutant of Nup107, respectively (related to Fig. 1).

**Additional file 3: Fig. S2.** G9a/GLP methylate ATF7IP and ATF7IP2. **A–B** An *in vitro* methylation assays were performed as in Fig. 1e (related to Fig. 1).

**Additional file 4: Fig. S3.** Establishment of ATF7IP-rescued cell lines. Western blot analysis confirmed a comparative expression of 3xF-ATF7IP in the cell lines (related to Fig. 2).

**Additional file 5: Table S2.** List of selected peaks (related to Fig. 2a).

**Additional file 6: Fig. S4.** Representative SPR sensorgram (left) and affinity curves (right) of human G9a binding to immobilized human histone H3, ATF7IP and LIG1 peptide. G9a was injected over the sensor chip immobilized with (A) unmodified H3, (B) H3K9me1, (C) H3K9me2, (D) H3K9me3, (E) unmodified ATF7IP, (F) ATF7IP K16me1, (G) ATF7IP K16me2, (H) ATF7IP K16me3, (I) unmodified LIG1, (J) LIG1 K126me1, (K) LIG1 K126me2, and (L) LIG1 K126me3 peptides (related to Fig. 4).

**Additional file 7: Table S3.** List of ATF7IPme-binding proteins.

**Additional file 8: Fig. S5.** MPP8 contributes to the silencing of MSCV-GFP in mESCs. **A** Confirmation of *Mpp8* KO by western blot analysis. **B** MSCV-GFP expression was analyzed by flow cytometric analysis. Both *Mpp8* KO cell lines showed increased GFP expression. **C** RT-qPCR analysis was performed. GFP mRNA expression was normalized to Hprt expression and is shown relative to the level in WT cells. Data are mean ± SEM; *n* = 4, biological replicates. *P < 0.05 by unpaired Student’s *t* test (related to Fig. 6).

**Additional file 9: Table S4.** Primers and plasmids.
