# Supplementary figures and images for: Tri-methylation of ATF7IP by G9a/GLP recruits the chromodomain protein MPP8
Source: Epigenetics Chromatin. 2018 Oct 4;11:56. doi: 10.1186/s13072-018-0231-z (PMC6172828; doi:10.1186/s13072-018-0231-z)

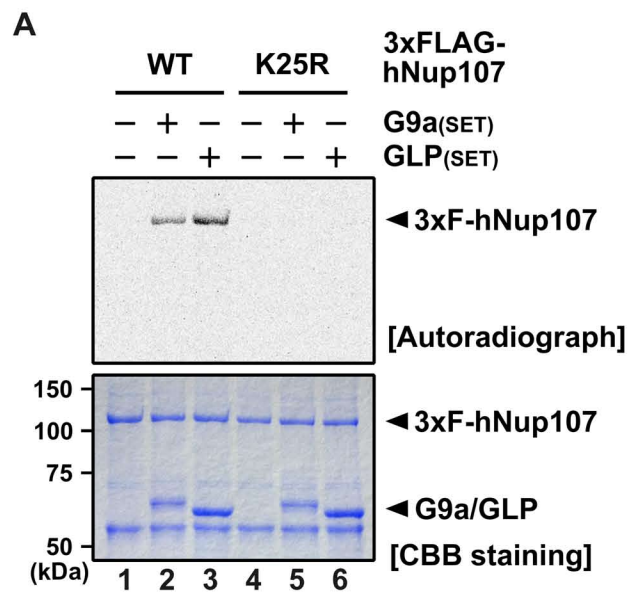

**Figure S1**

Supplement: Supplementary file 2 — Additional file 2: Fig. S1. An in vitro methylation assay with 3xFLAG-tagged hNup107 protein, GST-tagged SET-domain of G9a or GLP, and 14C-labelled SAM. 3xFLAG-tagged hNup107 was expressed in and purified from HEK293 cells transfected with the expression vector. An incorporation of the methyl-moiety was detected as an autoradiographic signal. WT and KR indicate wild-type and K25R mutant of Nup107, respectively (related to Fig. 1). [file 13072_2018_231_MOESM2_ESM.pdf]

**A**

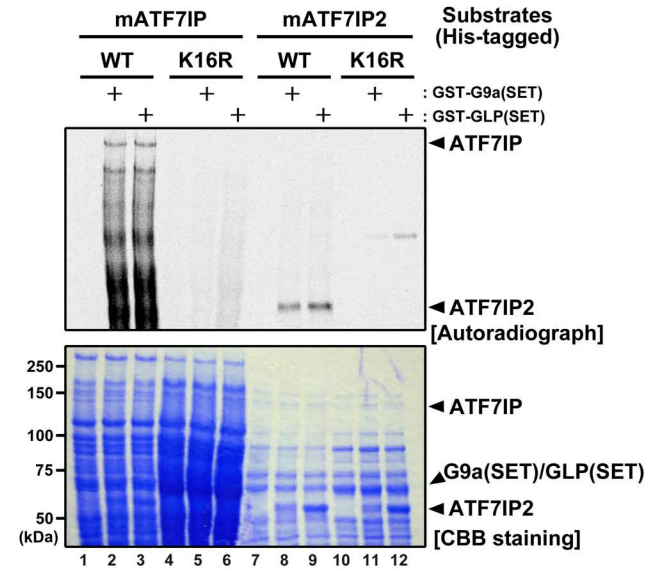

**B**

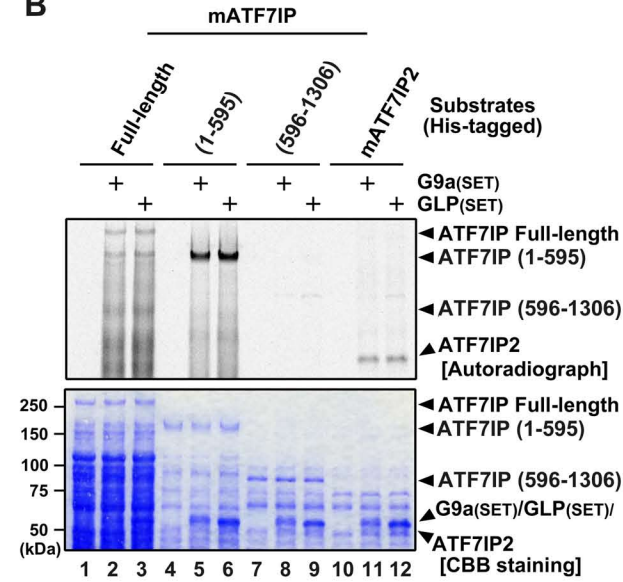

**Figure S2**

Supplement: Supplementary file 3 — Additional file 3: Fig. S2. G9a/GLP methylate ATF7IP and ATF7IP2. A–B An in vitro methylation assays were performed as in Fig. 1e (related to Fig. 1). [file 13072_2018_231_MOESM3_ESM.pdf]

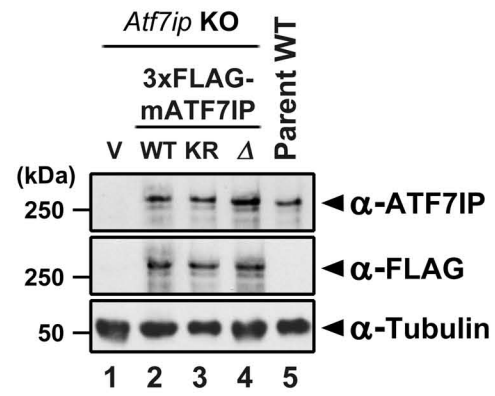

Figure S3

Supplement: Supplementary file 4 — Additional file 4: Fig. S3. Establishment of ATF7IP-rescued cell lines. Western blot analysis confirmed a comparative expression of 3xF-ATF7IP in the cell lines (related to Fig. 2). [file 13072_2018_231_MOESM4_ESM.pdf]

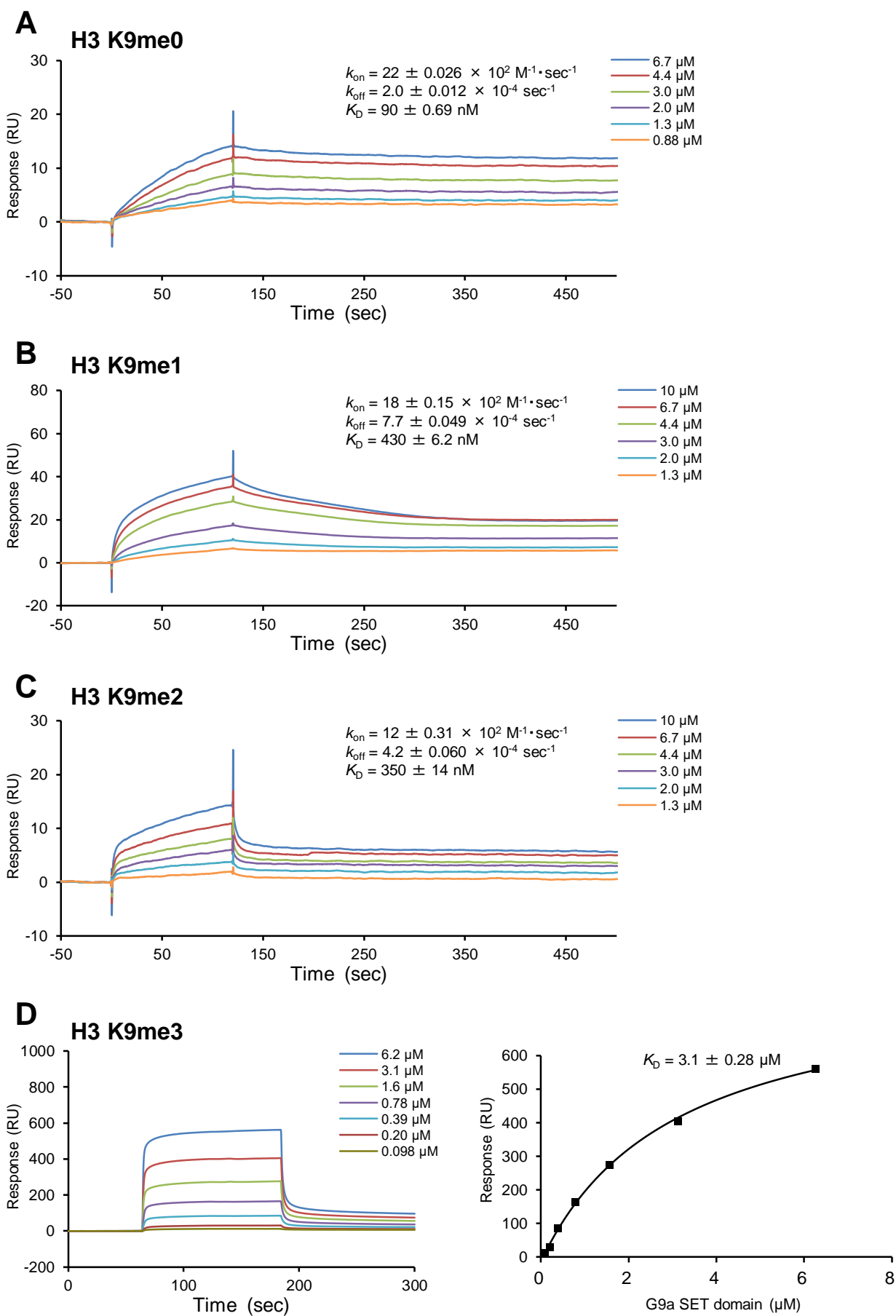

**Figure S4 (1)**

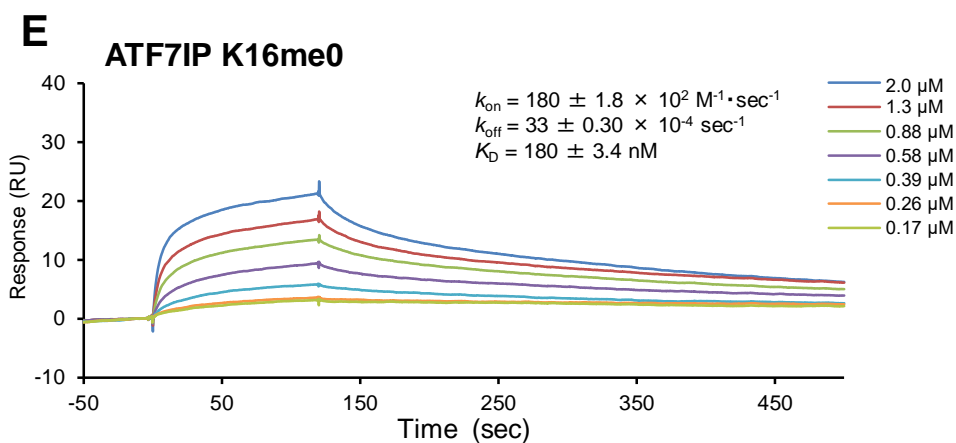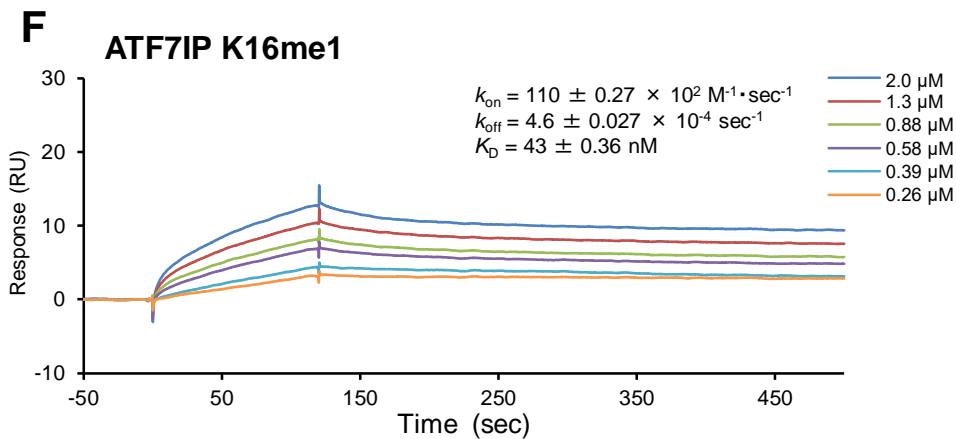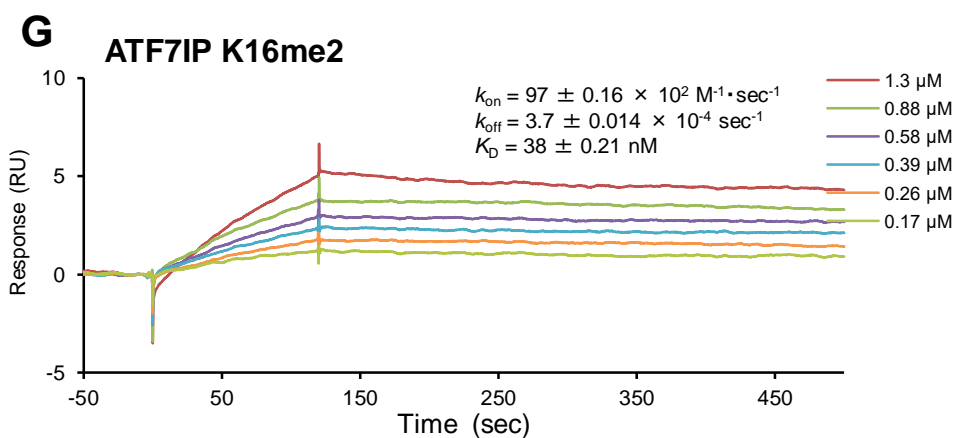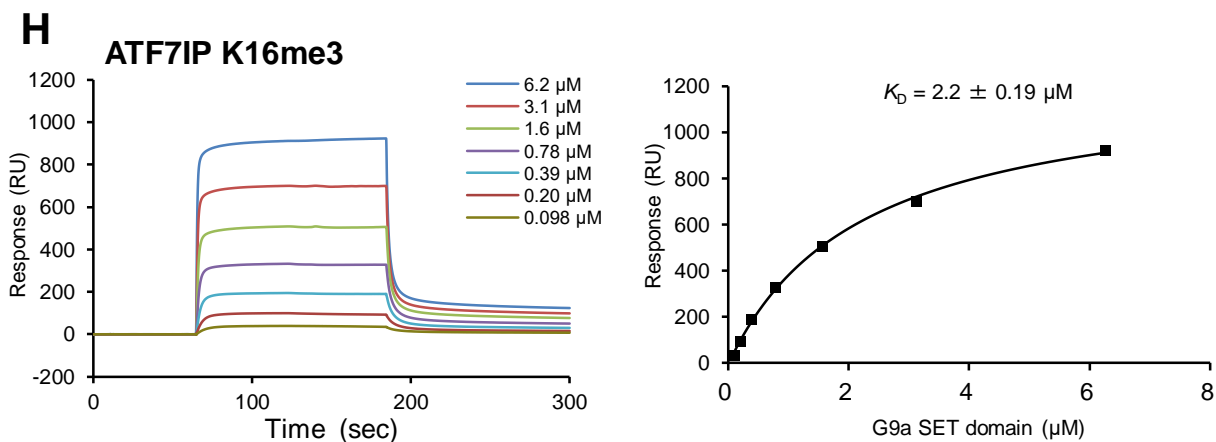

**Figure S4 (2)**

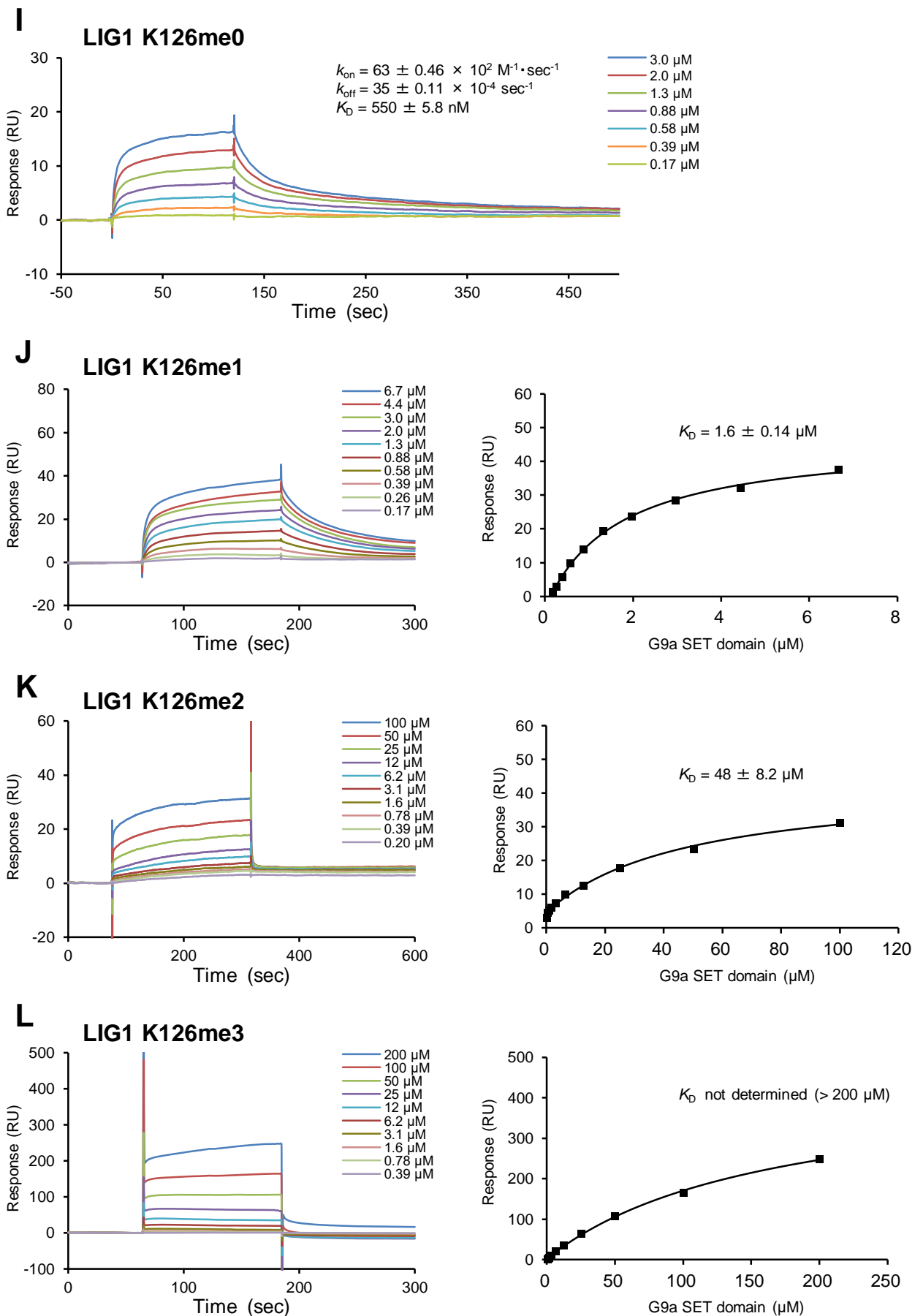

**Figure S4 (3)**

Supplement: Supplementary file 6 — Additional file 6: Fig. S4. Representative SPR sensorgram (left) and affinity curves (right) of human G9a binding to immobilized human histone H3, ATF7IP and LIG1 peptide. G9a was injected over the sensor chip immobilized with (A) unmodified H3, (B) H3K9me1, (C) H3K9me2, (D) H3K9me3, (E) unmodified ATF7IP, (F) ATF7IP K16me1, (G) ATF7IP K16me2, (H) ATF7IP K16me3, (I) unmodified LIG1, (J) LIG1 K126me1, (K) LIG1 K126me2, and (L) LIG1 K126me3 peptides (related to Fig. 4). [file 13072_2018_231_MOESM6_ESM.pdf]

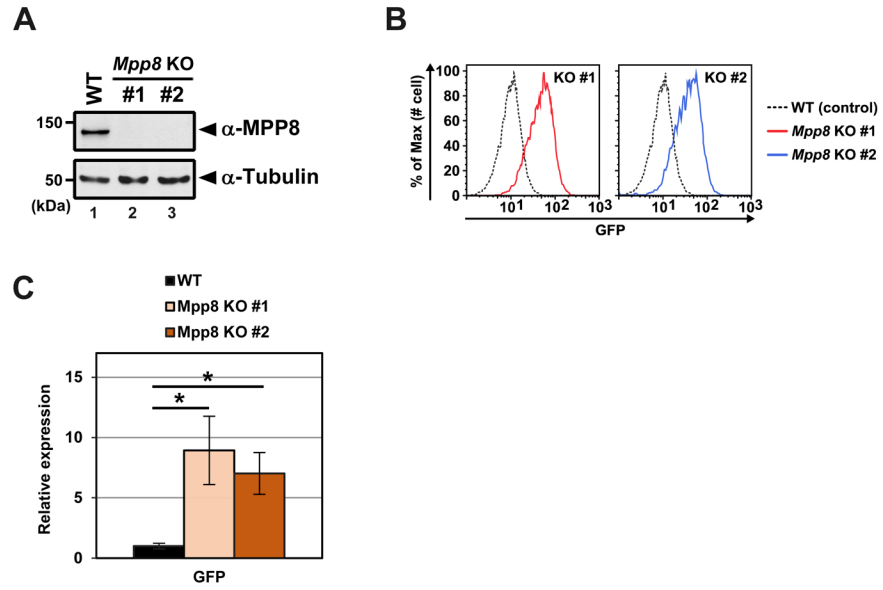

Figure S5

Supplement: Supplementary file 8 — Additional file 8: Fig. S5. MPP8 contributes to the silencing of MSCV-GFP in mESCs. A Confirmation of Mpp8 KO by western blot analysis. B MSCV-GFP expression was analyzed by flow cytometric analysis. Both Mpp8 KO cell lines showed increased GFP expression. C RT-qPCR analysis was performed. GFP mRNA expression was normalized to Hprt expression and is shown relative to the level in WT cells. Data are mean ± SEM; n = 4, biological replicates. *P < 0.05 by unpaired Student’s t test (related to Fig. 6). [file 13072_2018_231_MOESM8_ESM.pdf]
